# Temporary Upregulation of Nrf2 by Naringenin Alleviates Oxidative Damage in the Retina and ARPE-19 Cells

**DOI:** 10.1155/2021/4053276

**Published:** 2021-11-17

**Authors:** Wenpei Chen, Yuxin Ye, Zhongrui Wu, Junli Lin, Yiting Wang, Qi Ding, Xinrong Yang, Wei Yang, Bingqing Lin, Baoqin Lin

**Affiliations:** ^1^School of Pharmaceutical Sciences, Guangzhou University of Chinese Medicine, Guangzhou, Guangdong 510006, China; ^2^Guangdong Lewwin Pharmaceutical Research Institute Co., Ltd., Guangdong Provincial Key Laboratory of Drug Non-clinical Evaluation and Research, TCM Non-clinic Evaluation Branch of National Engineering Research Center for Modernization of Traditional Chinese Medicine, Guangdong Provincial Center for Ophthalmic Drug Creation and Evaluation Engineering Technology, Guangzhou, Guangdong 510990, China; ^3^College of Mathematics and Statistics, Shenzhen University, Shenzhen, Guangdong 518060, China

## Abstract

Dry age-related macular degeneration (dAMD) is a chronic degenerative ophthalmopathy that leads to serious burden of visual impairment. Antioxidation in retinal pigment epithelium (RPE) cells is considered as a potential treatment for dAMD. Our previous studies have showed that naringenin (NAR) protects RPE cells from oxidative damage partly through SIRT1-mediated antioxidation. In this study, we tested the hypothesis that the Nrf2 signaling is another protective mechanism of NAR on dAMD. NaIO_3_-induced mouse retinopathy and ARPE-19 cell injury models were established. Immunochemical staining, immunofluorescence, and western blotting were performed to detect the protein expressions of Nrf2 and HO-1. In addition, ML385 (activity inhibitor of Nrf2) and zinc protoporphyrin (ZnPP, activity inhibitor of HO-1) were applied to explore the effect of NaIO_3_ or NAR. The results showed that NAR increased the protein expressions of Nrf2 and HO-1 in the retinas in mice exposed to NaIO_3_ at the early stage. NAR treatment also resulted in a stronger activation of Nrf2 at the early stage in NaIO_3_-treated ARPE-19 cells. Moreover, inhibition of HO-1 by ZnPP weakened the cytoprotective effect of NAR. The constitutive accumulation and activation of Nrf2 induced by NaIO_3_ led to the death of RPE cells. However, NAR decreased the protein expressions of Nrf2 and HO-1 towards normal level in the mouse retinas and ARPE-19 cells exposed to NaIO_3_ at the late stage. Our findings indicate that NAR protects RPE cells from oxidative damage via activating the Nrf2 signaling pathway.

## 1. Introduction

Age-related macular degeneration (AMD) is a progressive chronic ophthalmopathy with aging and eventually results in serious visual impairment. Among patients with AMD, dry AMD (dAMD) accounts for 85%-90%, and some of which may develop into geographic atrophy (GA) and/or wet AMD (wAMD). In dAMD, retinal pigment epithelium (RPE) is accepted as the primary lesion, and photoreceptors are the second one that contributes to visual impairment [1, 2].

Oxidative stress is considered as a central contributor to the progress of dAMD. Prooxidative factors such as aging, smoking, and sunlight lead to the oxidative insult in RPE cells, manifesting as the formation of deposit and drusen between the RPE and Bruch membrane (BrM). The accumulations of deposit and drusen boost the thickness of BrM. Besides causing the death of RPE, oxidative stress compels RPE to detach from BrM and then makes RPE losing function. In dAMD patients, RPE cells detached from BrM are going forward into the nerve fiber layer or backward into the BrM [3, 4]. The displacement of RPE may be the result of epithelium-to-mesenchymal transition. As a consequence, RPE cells may survive at the cost of loss of function. The death and dysfunction of RPE result in poor nutrient supply of photoreceptors, which causes the atrophy of photoreceptors gradually [2]. Therefore, antioxidation targeting to RPE cells is a potential treatment for dAMD.

Naringenin (NAR), 4′,5,7-trihydroxyflavanone, is rich in fruits such as grapefruit and citrus. In our previous report, topical administration of NAR eye drops improved retinal function and structure in murine with retina degeneration induced by NaIO_3_ [5, 6] or N-methyl-N-nitrosourea (MNU) [7]. Antioxidant activity of NAR on age-related neurodegenerative diseases has aroused special attention [8]. We found that NAR protects RPE cells from NaIO_3_-induced oxidative damage through upregulation of SIRT1 [6]. However, inhibition of SIRT1 decreased the protective effect of NAR on cell viability by 69.2% in ARPE-19 cells incubated with NaIO_3_, which suggests that other protective pathways of NAR may exist.

Nuclear factor erythroid 2-related factor 2 (Nrf2) is an essential regulator of redox homeostasis. Under normal condition, Nrf2 is suppressed by Kelch-like ECH-associated protein 1 (Keap1) and anchored in the cytoplasm. While under oxidative stress, Nrf2 is dissociated from Keap1 and transits to the nucleus [9]. Nrf2 aggregates in the nucleus and then heterodimerizes with small Maf proteins and binds to the antioxidant response element (ARE), which mediates the transcription of various antioxidant enzymes, such as superoxide dismutase (SOD), glutathione peroxidase (GPx), glutathione reductase (GR), thioredoxin reductase (TrxR), peroxiredoxin (Prx), hemeoxygenase-1 (HO-1), and NAD(P)H:quinone oxidoreductase 1 (NQO1) [10]. Several evidences have demonstrated that the expression of Nrf2 and its activity decrease during aging, meaning Nrf2 is an important regulator in cell senescence [11–13]. Recent studies have shown that aged Nrf2^−/−^ mice develop dAMD-like pathology presenting as vacuolation of RPE, accumulation of lipofuscin, drusen-like deposits, and sub-RPE deposits of inflammatory proteins [14, 15]. Upregulation of Nrf2 rescues retinal function and protects retinal cells from oxidative damage [16–18]. Therefore, upregulation of Nrf2 is a potential antioxidant strategy for dAMD.

Therefore, in this study, we investigated whether Nrf2/HO-1-mediated antioxidant mechanism was involved in the protective effect of NAR in NaIO_3_-treated mice *in vivo* and ARPE-19 cells *in vitro*.

## 2. Materials and Methods

This study used the method of Chen et al., and the method description partly reproduced their wording [6].

### 2.1. Reagents

NAR (purity > 98% by high-performance liquid chromatography), NaIO_3_, and 4′,6-diamidino-2-phenylindole dihydrochloride (DAPI) were bought from Sigma-Aldrich (MO, USA). Zinc protoporphyrin (ZnPP) and ML385 were obtained from Meilun Biotechnology Co., Ltd. (Dalian, China) and TargetMol (Shanghai, China), respectively. DMEM/F-12 nutrient mixture and foetal bovine serum (FBS) were from Gibco (CA, USA) and BI (Kibbutz Beit HaEmek, Israel), respectively. Penicillin-streptomycin mixed antibiotic was obtained from Solarbio Life Sciences (Beijing, China). Antibodies against Nrf2, HO-1, and histone H3 were obtained from Abcam (Cambridge, UK). Antibody against glyceraldehyde-3-phosphate dehydrogenase (GAPDH) was from CST (MA, USA). Horseradish peroxidase- (HRP-) conjugated anti-rabbit and anti-mouse IgG were from Jackson ImmunoResearch (MA, USA). Goat anti-rabbit IgG conjugated to Alexa Fluor 594 was from Abbkine (CA, USA). RIPA and phenyl methyl sulfonyl fluoride (PMSF) were purchased from CWBIO (Beijing, China). BCA kit was obtained from Generay (Shanghai, China). Polyvinylidene difluoride (PVDF) membranes and commercial oxidized protein detection kit were purchased from Millipore (MA, USA). Diaminobenzidine (DAB) and WST-8 were from ZSGB-BIO (Beijing, China) and Dojindo Laboratories (Kumamoto, Japan), respectively. Nuclear and cytoplasmic protein extraction kit was bought from Beyotime (Shanghai, China). Hematoxylin and 4′,6-diamidino-2-phenylindole (DAPI) were obtained from MXB (Fuzhou, China) and Sigma-Aldrich (MO, USA), respectively.

### 2.2. Animals

Male and female Kunming mice weighing 22-24 g were provided by the Experimental Animal Center, Guangzhou University of Chinese Medicine (approved no. SCXK 2018-0034). The animals had free access to a standard diet and water and were housed in a room at 24.0 ± 0.5°C and with a 12 h/12 h cyclic lighting schedule. Experiments were carried out in compliance with the Animal Ethics Committee of Guangzhou University of Chinese Medicine (Approved No. 20190715001).

### 2.3. NaIO_3_-Induced Retinal Degeneration and Animal Treatment

Mice were randomly divided into three groups: normal group, model group, and 1% NAR group. Normal mice were injected with saline intraperitoneally, while others were intraperitoneally injected with a single dose of 25 mg/kg NaIO_3_. All the eyes of mice in the normal and model groups were topically administered with vehicle solution. All the eyes of mice in the 1% NAR group were topically administered with one drop of 1% NAR triple daily from 1 d before to 2 d or 10 d after injection of NaIO_3_. At 2 d or 10 d after NaIO_3_ injury, all the eyes were removed for fixing with solution containing glacial acetic acid, formaldehyde, 95% ethanol, and distilled water in a ratio of 1 : 2 : 5 : 3 or collecting the retinas. Protein expressions of Nrf2 and HO-1 in the retinas were detected with immunochemical staining and western blotting.

### 2.4. Immunochemical Staining

Immunochemical staining procedure was carried out as our previous report [6]. Briefly, deparaffinized sections were blocked, underwent antigen retrieval, and then incubated. Sections were incubated with primary antibodies against Nrf2 (1 : 250) and HO-1 (1 : 600) at 4°C overnight. After washing, sections were then incubated with HRP-conjugated anti-rabbit or anti-mouse IgG (1 : 400) for 1 h. After washing, sections were incubated with DAB and slightly stained with hematoxylin. Immunostaining images were taken by a fluorescence microscope (BX53F; OLYMPUS, Tokyo, Japan).

### 2.5. Cell Culture

ARPE-19 is a kind of human RPE cell line that obtained from China Center for Type Culture Collection (Wuhan, China). Cells were cultured in complete medium containing DMEM/F-12 medium and 10% FBS and antibiotics (100 U/mL penicillin and 100 *μ*g/mL streptomycin) at 37°C in a humidified atmosphere of 5% CO_2_.

### 2.6. Cell Viability of NaIO_3_-Treated ARPE-19 Cells

ARPE-19 cells were seeded into 96-well plates and cultured in complete medium overnight. Cells were cotreated with NaIO_3_, NAR, ZnPP, or ML385 for 24 h or 48 h. Cells were then incubated with fresh complete medium containing 10% WST-8 for 1.5 h. Absorbance at 450 nm was recorded by a microplate reader (Multiskan GO; Thermo Scientific, USA). The final data were expressed as percentage relative to the normal group.

### 2.7. Immunofluorescence Analysis

Cells were seeded on glass coverslips and cultured in 24-well plates overnight. After incubating with NaIO_3_ and ML385, cells were fixed with precold 4% paraformaldehyde for 15 min, permeabilized with 1% Triton for 20 min, and then blocked with 10% goat serum for 30 min. Cells were then incubated with antibody against Nrf2 (1 : 400) at 4°C overnight. Thereafter, cells were incubated with goat anti-rabbit IgG conjugated to Alexa Fluor 549 for 1 h. Sections were then stained with DAPI for 5 min. Fluorescence images were captured with a confocal fluorescence microscope (LSM800; Carl Zeiss, Jena, Germany).

### 2.8. Extraction of Protein

Retinal samples of mice taken from -80°C were lysed with solution containing RIPA buffer and PMSF in a ratio of 99 : 1 on ice for 30 min. Supernatant was collected to obtain total protein after centrifugation at 14,000 × *g* for 10 min at 4°C. At the end of the culture period, cells were rinsed with cold PBS and lysed. Supernatants were collected after centrifugation to obtain total protein. Nuclear protein was collected according to the manufacturer's instruction of a nuclear and cytoplasmic protein extraction kit.

### 2.9. Western Blotting Analysis

Protein samples were quantified with BCA method. Denatured protein samples were separated by 10% polyacrylamide gels electrophoresis and transferred onto PVDF membranes. After rinsing, the membranes were blocked for 2 h and probed with primary antibodies against Nrf2 (1 : 1000), HO-1 (1 : 2000), histone H3 (1 : 1000), or GAPDH (1 : 1000) at 4°C overnight. Afterwards, the membranes were incubated with HRP-conjugated anti-mouse or anti-rabbit IgG (1 : 5000) for 1 h. Immunoreactive protein bands were visualized with a chemiluminescence apparatus (5200CE; Tanon, Shanghai, China) and analyzed with ImageJ (NIH, MD, USA).

### 2.10. Measurement of Intracellular ROS

The program of detecting ROS was according to our previous report [6]. Cells were seeded into 35 mm dishes and cultured overnight. Then, cells were incubated with NaIO_3_ and ML385 for 24 h. After washing, cells were incubated with medium containing 2.5 *μ*M H_2_DCFDA (Jiancheng Bioengineering Institute, Nanjing, China) for 30 min. Images were collected using a fluorescence microscope.

### 2.11. Measurement of Carbonyl Protein

The expression of carbonyl protein was measured using a commercial oxidized protein detection kit. The program of detecting carbonyl protein was according to our previous report [6]. Cells were seeded on glass coverslips and cultured in 24-well plates overnight. Then, cells were incubated with NaIO_3_ and ML385 for 24 h. Cells were fixed with precold methanol, then incubated with 2,4-dinitrophenylhydrazine (DNP) solution, blocked with blocking buffer, and finally incubated with biotinylated anti-DNP antibody at 4°C overnight. Then, cells were incubated with streptavidin-Cy3 and then DAPI. Images were collected using a confocal fluorescence microscope.

### 2.12. Statistical Analysis

All data were represented as the mean ± standard error of mean (SEM) and analyzed by Statistical Package for the Social Sciences version 21.0 (SPSS 21.0). Statistical comparisons were performed using Student's *t*-test, one-way analysis of variance (ANOVA), or Mauchly's test of sphericity with Tukey's test. *P* < 0.05 was assumed to be significant.

## 3. Results

### 3.1. NAR Eye Drops Protect Retinal Morphology from NaIO_3_-Induced Injury in Mice

**Figure 1:**
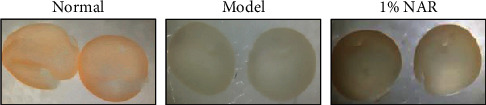
NAR eye drops improved the retinal morphology from NaIO_3_-induced injury in mice.

As shown in Figure 1, when the retinas were detached from the eyes, we found that the retinas of normal mice were light yellow and manifested as a film of half cup while the retinas damaged by NaIO_3_ were pale, thin, and friable. Treatment of 1% NAR eye drops for 10 d visibly improved the appearance of the retinas.

### 3.2. Localization of Nrf2 and HO-1 in the Mouse Retinas

**Figure 2:**
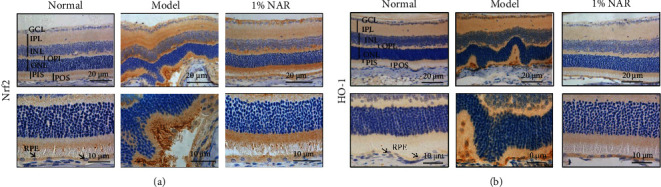
Localization of Nrf2 and HO-1 proteins in the mouse retinas after NaIO_3_ exposure and NAR treatment. (a) Representative images of Nrf2 immunostaining after 10 d of NaIO_3_ stimulation. (b) Representative images of HO-1 immunostaining after 10 d of NaIO_3_ stimulation. GCL: ganglion cell layer; IPL: inner plexiform layer; INL: inner nuclear layer; OPL: outer plexiform layer; ONL: outer nuclear layer; PIS: photoreceptor inner segment; POS: photoreceptor outer segment; RPE: retinal pigment epithelium.

As shown in Figures 2(a) and 2(b), Nrf2 and HO-1 proteins were less labeled in the normal retina. After NaIO_3_ stimulation, Nrf2 and HO-1 proteins were increased in the ganglion cell layer (GCL), inner plexiform layer (IPL), outer plexiform layer (OPL), photoreceptor inner segment (PIS), and especially photoreceptor outer segment (POS) and RPE. Nrf2 and HO-1 protein levels in the mentioned layers, particularly in the POS and RPE, were lower in NAR-treated mice.

### 3.3. NAR Regulates the Protein Expressions of Nrf2 and HO-1 at Early and Late Stages in the Retinas of Mice with NaIO_3_-Induced Retinopathy

**Figure 3:**
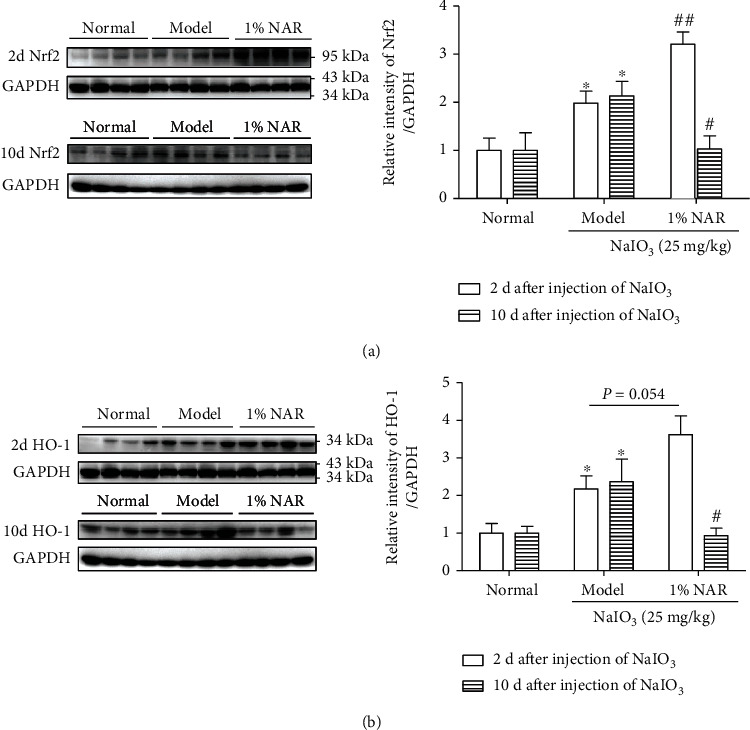
The effect of NAR on protein expressions of Nrf2 and HO-1 in the retinas of NaIO_3_-induced injury mice. (a) Protein expression of Nrf2 by western blotting analysis. (b) Protein expression of HO-1 by western blotting analysis. Data were represented as the mean ± SEM, *n* = 4 for 2 d after NaIO_3_ stimulation and *n* = 7 for 10 d after NaIO_3_ stimulation. ^∗^*P* < 0.05 vs. corresponding normal group and ^#^*P* < 0.05 and ^##^*P* < 0.01 vs. corresponding model group (Student's *t*-test).

The protein expressions of Nrf2 and HO-1 in the retinas of model rats were markedly increased at 2 d and 10 d after NaIO_3_ injection (Figures 3(a) and 3(b)). Compared to the model group, NAR treatment further increased Nrf2 and HO-1 protein levels by 122% and 145% after 2 d of NaIO_3_ injury, respectively. However, NAR treatment decreased both Nrf2 and HO-1 protein expressions towards normal levels after 10 d of NaIO_3_ injury.

### 3.4. NAR Regulates Nrf2 Expression at Early and Late Stages in NaIO_3_-Stimulated ARPE-19 Cells

**Figure 4:**
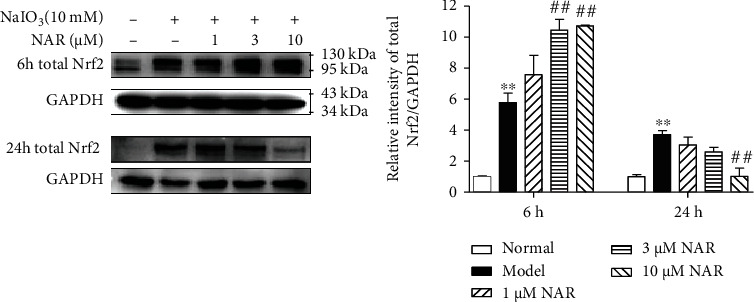
The effect of NAR on Nrf2 protein expression in NaIO_3_-stimulated ARPE-19 cells. Data were expressed as the mean ± SEM, *n* = 3. ^∗∗^*P* < 0.01 vs. normal group and ^##^*P* < 0.01 vs. model group (Tukey's test).

We next investigated the Nrf2 signaling pathway regulated by NAR in NaIO_3_-stimulated ARPE-19 cells. We first assayed Nrf2 protein expression in ARPE-19 cells incubated with 10 mM NaIO_3_ and NAR for 6 h and 24 h. NaIO_3_ exposure for 6 h significantly increased the expression of Nrf2 about 5.5-fold higher than that in normal ARPE-19 cells (Figure 4). The protein level of Nrf2 in cells treated with 3 and 10 *μ*M NAR for 6 h was increased by about 9.5-fold as compared with that of normal cells. Interestingly, 10 *μ*M NAR decreased Nrf2 towards normal level after 24 h treatment.

### 3.5. NAR Activates Nrf2 Much Earlier and Stronger in NaIO_3_-Treated ARPE-19 Cells

**Figure 5:**
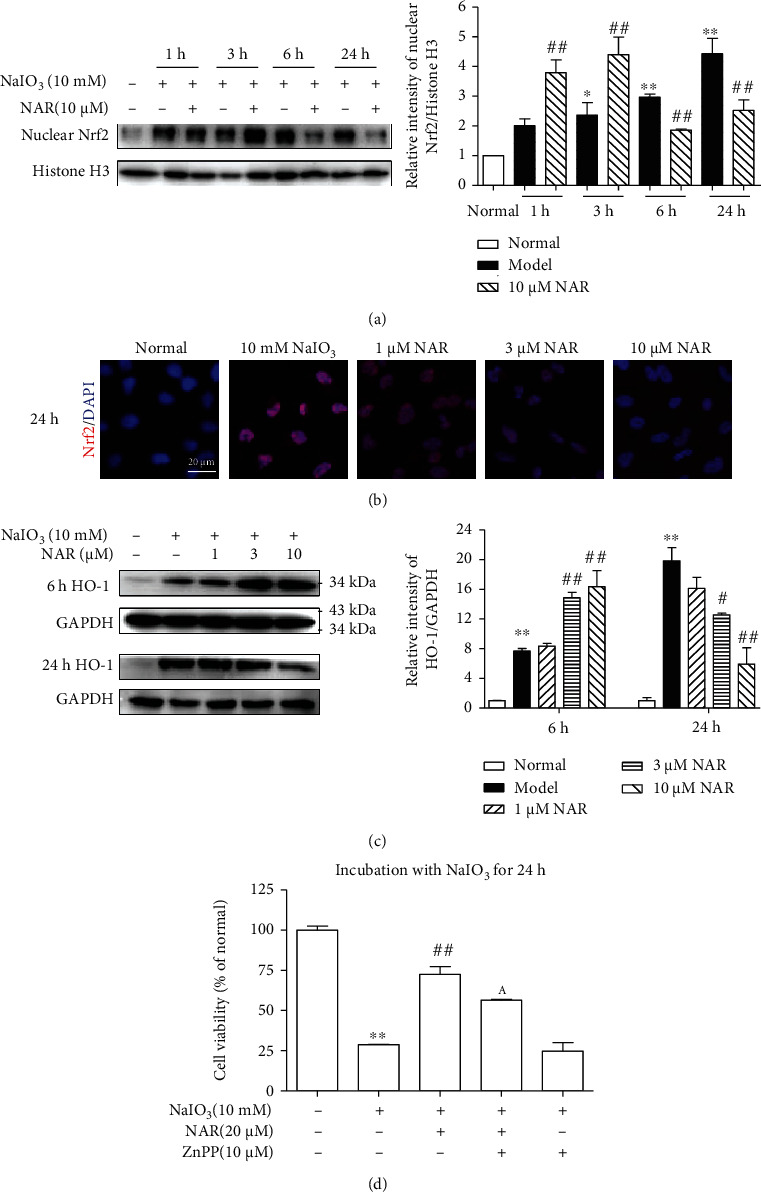
The effect of NAR on Nrf2 activation after NaIO_3_-induced damage in ARPE-19 cells. (a) Nuclear protein expression of Nrf2 after treatment for 1-24 h (Mauchly's test of sphericity with Tukey's test). (b) Representative fluorescence images of Nrf2 after 24 h treatment. (c) Protein expression of HO-1 after 6 h and 24 h treatment (Tukey's test). (d) Viability of cells cotreated with NAR and/or ZnPP (Tukey's test). Data were expressed as the mean ± SEM, *n* = 3 − 6. ^∗^*P* < 0.05 and ^∗∗^*P* < 0.01 vs. normal group, ^#^*P* < 0.05 and ^##^*P* < 0.01 vs. model group, and ^A^*P* < 0.05 vs. NAR group.

We also analyzed the dynamic activation of Nrf2 to clarify whether NAR stimulated Nrf2 activation. Firstly, we analyzed the protein expression of Nrf2 in the nuclei by western blotting and immunostaining. As shown in Figure 5(a), nuclear Nrf2 level continuously increased during incubation with NaIO_3_ from 3 h to 24 h. Compared with the NaIO_3_ group, NAR increased nuclear Nrf2 protein content to almost twofold in the early stage of NaIO_3_-induced damage (1-3 h) and then significantly decreased nuclear Nrf2 protein in the late stage (6-24 h). Immunostaining result showed that Nrf2 protein expression in the nuclei of cells was decreased after 24 h treatment of NAR (Figure 5(b)).

Then, HO-1 protein expression was determined after 6 h and 24 h incubation with 10 mM NaIO_3_. Compared with the NaIO_3_ group, HO-1 protein expression in the NAR group was increased after 6 h treatment and then decreased after 24 h (Figure 5(c)). In addition, ZnPP, an HO-1 inhibitor, significantly suppressed the protective effect of NAR by 36.9% (Figure 5(d)).

### 3.6. The Abnormal Nrf2 Signaling Pathway Induced by NaIO_3_ Promotes the Death of ARPE-19 Cells

**Figure 6:**
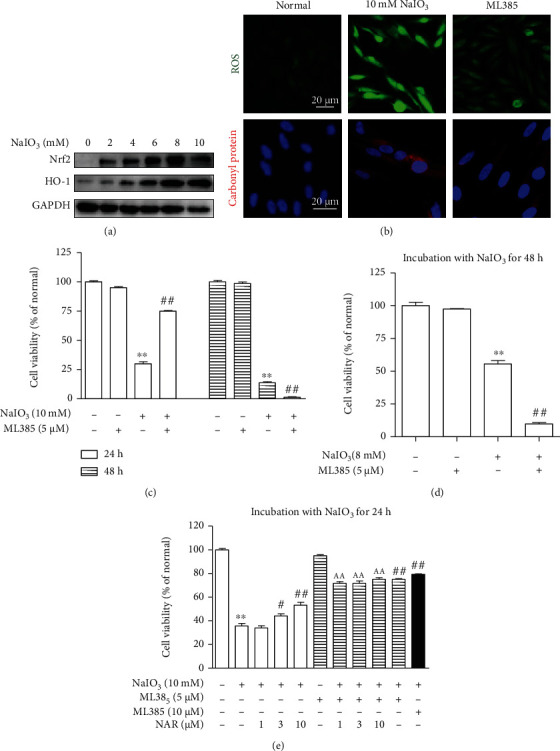
Overexpression of Nrf2 induced by NaIO_3_ promoted the death of ARPE-19 cells. (a) Protein expressions of Nrf2 and HO-1 in ARPE-19 cells stimulated by NaIO_3_ for 24 h. (b) Representative fluorescence images of ROS and carbonyl protein in ARPE-19 cells cotreated with 10 mM NaIO_3_ and ML385 for 24 h. (c) The viability of cells cotreated with 10 mM NaIO_3_ and ML385 for 24 h and 48 h. (d) The viability of cells cotreated with 8 mM NaIO_3_ and ML385 for 48 h. (e) The viability of cells cotreated with 10 mM NaIO_3_, ML385, and/or NAR for 24 h. Data were expressed as the mean ± SEM, *n* = 4. ^∗∗^*P* < 0.01 vs. normal group, ^##^*P* < 0.01 vs. model group, and ^AA^*P* < 0.01 vs. corresponding NAR group (Tukey's test).

In animal and cell models, we found that high protein expressions of Nrf2 and HO-1 were induced by NaIO_3_. To evaluate the effect of increased Nrf2, ML385, an inhibitor of Nrf2 activation, was used. NaIO_3_ increased protein expressions of Nrf2 and HO-1 in a concentration-dependent manner (Figure 6(a)). Interestingly, 24 h treatment with ML385 reduced the levels of ROS and carbonyl protein (Figure 6(b)). Moreover, 24 h treatment with ML385 improved viability by 45.1% in 10 mM NaIO_3_-stimulated cells (Figure 6(c)), but 48 h treatment significantly accelerated the death of cells treated with 8 mM (Figure 6(d)) or 10 mM NaIO_3_ (Figure 6(c)). In addition, ML385 in combination of different concentrations of NAR for 24 h showed the same protective effects on cells injured by NaIO_3_ (Figure 6(e)).

## 4. Discussion

Our previous study showed that NAR significantly improved retinal function and morphology in NaIO_3_-treated mice and protected ARPE-19 cells partly via SIRT1-mediated antioxidation [6]. Here, we revealed that NAR activates the Nrf2 signaling pathway to protect RPE cells and the retinas from NaIO_3_-induced oxidative damage in the early stage.

Nrf2 is an essential regulator of redox homeostasis. However, Nrf2 expression and activity decrease with aging, which means the burden of oxidative stress gradually increases during lifetime [11–13]. Recent researches have shown that Nrf2 deficiency results in RPE cell damage and ocular pathology similar to human dAMD [14, 15], meaning that Nrf2 is a target for the treatment of dAMD. In response to weak oxidative stress, Nrf2 increases and induces antioxidant genes such as HO-1 and NQO1 to protect cells [19, 20]. We found that Nrf2 and HO-1 protein expressions were increased and mainly localized in POS and RPE in NaIO_3_-stimulated mice (Figures 2 and 3), which indicates that RPE is the major location to suffer oxidative insult from NaIO_3_. The upregulation of Nrf2 protein expression and activity was also observed in ARPE-19 cells stimulated by NaIO_3_ in vitro (Figures 4–6). These results indicate that NaIO_3_ induces oxidative stress and the adaptive protective response of Nrf2 is triggered in the early stage of oxidative damage in RPE cells [21].

However, if the stress is too severe, adaptive stress response may reach their boundary of activation and then lead to prohibit full recovery and provoke cell death [21]. It is reported that Nrf2 knockdown prevents cells from severe DNA damage [21], meaning high protein expression of Nrf2 is a risk factor when cells face to severe damage. Zucker et al. have demonstrated that according to different ROS levels, Nrf2 induces accumulation or removal of ROS via two separate regulatory circuits that are Klf9-dependent and HO-1/NOQ1-relative, respectively [19]. Upon severe oxidative stress, Klf9-dependent accumulation of ROS holds dominant position via repressing antioxidant enzymes such as TrxR2 and Prx6 to compel cell death [19, 20]. Our results showed that NaIO_3_ promoted the protein expression of Nrf2 in ARPE-19 cells in a concentration-dependent manner (Figure 6(a)), while inhibition of Nrf2 activation by ML385 promoted cell survival and reduced the levels of ROS and carbonyl protein (Figures 6(b) and 6(c)). These findings suggest that NaIO_3_ induces serious oxidative stress and Nrf2 amplifies oxidative stress.

The constitutive accumulation or activation of Nrf2 is also not beneficial for individual. The abnormal Nrf2 signaling pathway results in drug resistance in cancer cells [22, 23]. Several researches have shown sustained activation of the Nrf2/Keap1 redox sensing pathway, which ultimately led to age-dependent cardiac dysfunction and hypertrophic cardiomyopathy [24, 25]. In this study, with the relative persistence treatment by NAR, exorbitant protein expressions of Nrf2 and HO-1 were inhibited to normal level (Figures 2 and 3), which indicates that NAR may alleviate oxidative stress of the retina via inhibiting Nrf2/HO-1 in NaIO_3_-treated mice.

NAR was reported to ameliorate age-related neurodegenerative diseases through activating Nrf2 [26, 27]. Therefore, we next wanted to confirm whether NAR directly inhibits the Nrf2 signaling pathway to protect RPE or not. In the present study, NAR further increased protein expression of total Nrf2 at the early stage (within 6 h of NaIO_3_ damage) (Figures 3 and 5). Moreover, NAR treatment resulted in an earlier and stronger activation of Nrf2 in ARPE-19 cells than that in the NaIO_3_ alone group (Figures 5(a)–5(c)). These results indicate that NAR enhances the Nrf2 signaling pathway at the early stage of oxidative damage. Our finding is strengthened by a previous study which reveals that RS9, an Nrf2-specific activator, protects ARPE-19 cells from NaIO_3_-induced damage [28]. Activation of Nrf2 by RS9 accelerates the autophagic degradation of abnormal cytotoxic proteins in ARPE-19 cells after stimulating by NaIO_3_ within 6 h. In our study, the Nrf2 signaling pathway was activated strongly only within 1 h treatment with NAR, which indicates that NAR may protect ARPE-19 cells from oxidative stress through temporary upregulation of Nrf2.

Besides, we also observed that ZnPP, the special inhibitor of HO-1, suppressed the protective effect of NAR (Figure 5(d)), meaning that NAR protects RPE cells via the Nrf2/HO-1 pathway. Our result was similar to previous researches that 10 *μ*M ZnPP reversed the protective effect of NAR and pinocembrin against H_2_O_2_-induced cell death in SH-SY5Y cells [29, 30]. However, the effect of HO-1 inhibition was not consistent with the cytoprotective effect of Nrf2 inhibition. The contradictory results indicate that NAR protects RPE cells through enhancing the Nrf2/HO-1 pathway at the early stage and then decreases ROS, which may inhibit the Nrf2/Klf9 pathway and avoid cell death. Similar protective effects in cell viability were observed in ML385 and ML385 in combination with NAR in NaIO_3_-treated cells (Figure 6(e)). The reason may be that inhibition of Nrf2 activity inhibits not only the Nrf2/Klf9 and Nrf2/HO-1 pathways regulated but exorbitant ROS (NaIO_3_) but also the Nrf2/HO-1 pathway regulated by NAR. How NAR regulates the Nrf2 signaling needs to be further studied.

## 5. Conclusion

Taken together, NAR temporarily upregulated Nrf2 and HO-1 protein expressions and activated Nrf2 in NaIO_3_-treated ARPE-19 cells, suggesting that NAR prevents dAMD from oxidative stress through activating the Nrf2 signaling pathway.

## Data Availability

The data that support the findings of this study are available from the corresponding author upon reasonable request.

## Supplementary Materials

Supplementary Materials The protein expressions of Nrf2 and HO-1 in the retinas of each NaIO_3_-induced injury mice were showed in Figures S1(a) and S1(b). The total protein expressions of Nrf2 and HO-1 in ARPE-19 cells stimulated by NaIO_3_ were showed in Figures S1(c) and S1(e). The nuclear protein expressions of Nrf2 in ARPE-19 cells stimulated by NaIO_3_ were showed in Figure S1(d). Figure S1: protein expressions of Nrf2 and HO-1 in the mouse retinas or in ARPE-19 cells treated with NAR and/or NaIO_3_. (a) Protein expression of Nrf2 in the retinas of NaIO_3_-induced injury mice by western blotting analysis. (b) Protein expression of Nrf2 in the retinas of NaIO_3_-induced injury mice by western blotting analysis. (c) Protein expression of Nrf2 after 6 h and 24 h treatment of NAR and/or NaIO_3_ in ARPE-19 cells by western blotting analysis. (d) Nuclear protein expression of Nrf2 after treatment with NAR and/or NaIO_3_ for 1-24 h in ARPE-19 cells by western blotting analysis. (e) Protein expression of HO-1 after 6 h and 24 h treatment of NAR and/or NaIO_3_ in ARPE-19 cells by western blotting analysis.
