# Supplementary figures and images for: Temporary Upregulation of Nrf2 by Naringenin Alleviates Oxidative Damage in the Retina and ARPE-19 Cells
Source: Oxid Med Cell Longev. 2021 Nov 17;2021:4053276. doi: 10.1155/2021/4053276 (PMC8612781; doi:10.1155/2021/4053276)

Supplementary Figure 1


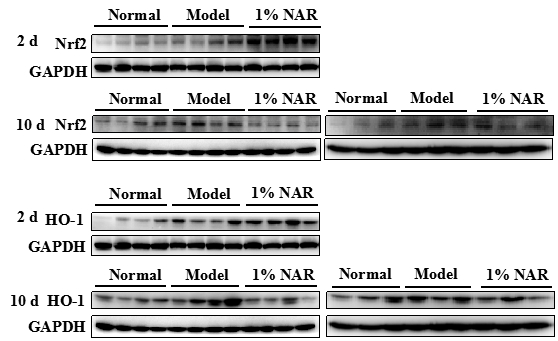


Supplementary Figure 2


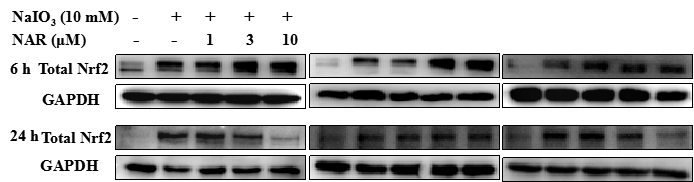


Supplementary Figure 3


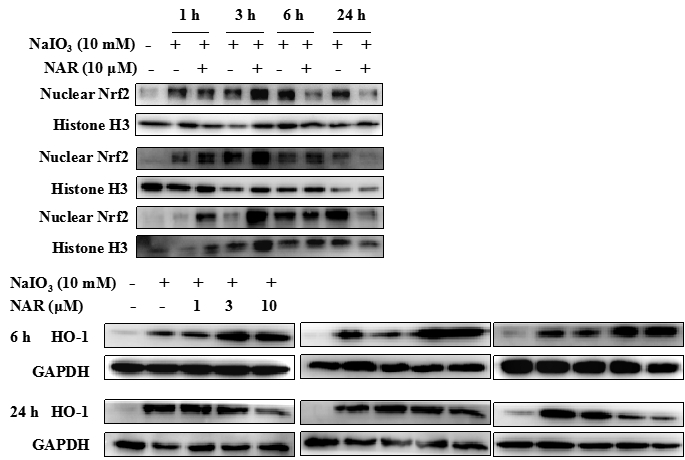

Supplement: Supplementary Materials — The protein expressions of Nrf2 and HO-1 in the retinas of each NaIO3-induced injury mice were showed in Figures S1(a) and S1(b). The total protein expressions of Nrf2 and HO-1 in ARPE-19 cells stimulated by NaIO3 were showed in Figures S1(c) and S1(e). The nuclear protein expressions of Nrf2 in ARPE-19 cells stimulated by NaIO3 were showed in Figure S1(d). Figure S1: protein expressions of Nrf2 and HO-1 in the mouse retinas or in ARPE-19 cells treated with NAR and/or NaIO3. (a) Protein expression of Nrf2 in the retinas of NaIO3-induced injury mice by western blotting analysis. (b) Protein expression of Nrf2 in the retinas of NaIO3-induced injury mice by western blotting analysis. (c) Protein expression of Nrf2 after 6 h and 24 h treatment of NAR and/or NaIO3 in ARPE-19 cells by western blotting analysis. (d) Nuclear protein expression of Nrf2 after treatment with NAR and/or NaIO3 for 1-24 h in ARPE-19 cells by western blotting analysis. (e) Protein expression of HO-1 after 6 h and 24 h treatment of NAR and/or NaIO3 in ARPE-19 cells by western blotting analysis. [file 4053276.f1.doc]
